# Lower Corticosteroid Skin Blanching Response Is Associated with Severe COPD

**DOI:** 10.1371/journal.pone.0091788

**Published:** 2014-03-12

**Authors:** Susan J. M. Hoonhorst, Nick H. T. ten Hacken, Adèle T. Lo Tam Loi, Leo Koenderman, Jan Willem J. Lammers, Eef D. Telenga, H. Marike Boezen, Maarten van den Berge, Dirkje S. Postma

**Affiliations:** 1 University of Groningen, University Medical Center Groningen, Department of Pulmonary Diseases, Groningen, the Netherlands; 2 University of Groningen, University Medical Center Groningen, GRIAC Research Institute, Groningen, the Netherlands; 3 University Medical Center Utrecht, Department of Respiratory Medicine, Utrecht, the Netherlands; 4 University of Groningen, University Medical Center Groningen, Department of Epidemiology, Groningen, the Netherlands; University of Tübingen, Germany

## Abstract

**Background:**

Chronic obstructive pulmonary disease (COPD) is characterized by chronic airflow limitation caused by ongoing inflammatory and remodeling processes of the airways and lung tissue. Inflammation can be targeted by corticosteroids. However, airway inflammation is generally less responsive to steroids in COPD than in asthma. The underlying mechanisms are yet unclear. This study aimed to assess whether skin corticosteroid insensitivity is associated with COPD and COPD severity using the corticosteroid skin blanching test.

**Methods:**

COPD patients GOLD stage I–IV (n = 27, 24, 22, and 16 respectively) and healthy never-smokers and smokers (n = 28 and 56 respectively) were included. Corticosteroid sensitivity was assessed by the corticosteroid skin blanching test. Budesonide was applied in 8 logarithmically increasing concentrations (0–100 μg/ml) on subject's forearm. Assessment of blanching was performed after 7 hours using a 7-point scale (normal skin to intense blanching). All subjects performed spirometry and body plethysmography.

**Results:**

Both GOLD III and GOLD IV COPD patients showed significantly lower skin blanching responses than healthy never-smokers and smokers, GOLD I, and GOLD II patients. Their area under the dose-response curve values of the skin blanching response were 586 and 243 vs. 1560, 1154, 1380, and 1309 respectively, p<0.05. Lower FEV_1_ levels and higher RV/TLC ratios were significantly associated with lower skin blanching responses (p = 0.001 and p = 0.004 respectively). GOLD stage I, II, III and IV patients had similar age and packyears.

**Conclusions:**

In this study, severe and very severe COPD patients had lower skin corticosteroid sensitivity than mild and moderate COPD patients and non-COPD controls with comparable age and packyears. Our findings together suggest that the reduced skin blanching response fits with a subgroup of COPD patients that has an early-onset COPD phenotype.

## Introduction

Chronic obstructive pulmonary disease (COPD) is characterized by chronic airflow limitation caused by ongoing inflammatory and remodeling processes of the airways and lung tissue [1]. Inflammation can be targeted with inhaled corticosteroids (ICS) and indeed ICS improve symptoms, quality of life and exacerbation rates in COPD [2], [3]. However, most studies did not find a reduction in lung function decline after long-term ICS treatment [4], indicating that the underlying process of disease progression was generally not effectively modified. Two studies showed beneficial effects of long-term ICS treatment on lung function decline [5], [6]. This suggests that a subgroup of COPD patients may be more sensitive to ICS, a finding recently corroborated by gene-expression profiling [7].

Notwithstanding above findings, it is clear that airway inflammation in COPD generally responds less well to corticosteroids than in asthma patients. However, it has not been fully established whether corticosteroid insensitivity is more pronounced in patients with more severe COPD. The TORCH study has shown that treatment with combined salmeterol and fluticasone reduced moderate-to-severe exacerbations and improved health status and forced expiratory volume in one second (FEV_1_) across all GOLD stages. Depending on the outcome parameters studied, the latter study showed additionally a better or equal ICS response in severe COPD compared to patients with milder disease [8].

Corticosteroid sensitivity can indirectly be assessed by the McKenzie skin blanching test [9]. In this test, corticosteroids are topically applied to the skin in 8 logarithmically increasing concentrations (0–100 μg/ml) and corticosteroid responsiveness can be determined by the degree of blanching of the skin. Asthma patients with airway obstruction unresponsive to corticosteroid treatment (i.e. a failure of FEV_1_ and peak expiratory flow to improve by at least 15% after 2-week corticosteroid treatment) have lower skin blanching scores than steroid responsive patients [10]. Furthermore, skin blanching scores are lower in smokers with asthma than in never-smokers with asthma and smokers without airway obstruction [11]. Additionally, we recently found that lower skin blanching scores were associated with a lower lung function in asthma patients [12]. So far, no data are available in COPD.

In this study we investigated the skin blanching test in patients with mild-to-very severe COPD and healthy never-smokers and long-term smokers. Our main objective was to ascertain whether COPD patients have lower corticosteroid sensitivity in the skin than never-smoking and heavy smoking healthy controls, as measured by the skin blanching response. Secondly, we investigated if the skin blanching response was associated with disease severity.

## Methods

### Study population

COPD patients and healthy individuals were recruited by advertisements and from hospital outpatient clinics. Mild-to-severe COPD patients were included with an FEV_1_/FVC<0.7 and stages I to IV according to the Global initiative for chronic Obstructive Lung Disease (GOLD) [1]. Never-smokers and current smokers without airway obstruction (FEV_1_/FVC>0.7) were included as healthy controls. Smokers had to have a smoking history of more than 20 packyears. Patients with a doctor's diagnosis of asthma or patients with alpha-1 antitrypsin deficiency were excluded. The study was approved by the medical ethics committees of University Medical Centers Groningen and Utrecht, The Netherlands. All subjects gave their written informed consent. The study is a multicenter study performed by University Medical Centers of Groningen and Utrecht, with trial register numbers NCT00807469 and NCT00850863 (www.clinicaltrials.gov) [13]. Healthy controls were obtained from the Norm study performed by University Medical Center Groningen, trial register number NCT00848406.

### Measurements

**Figure 1:**
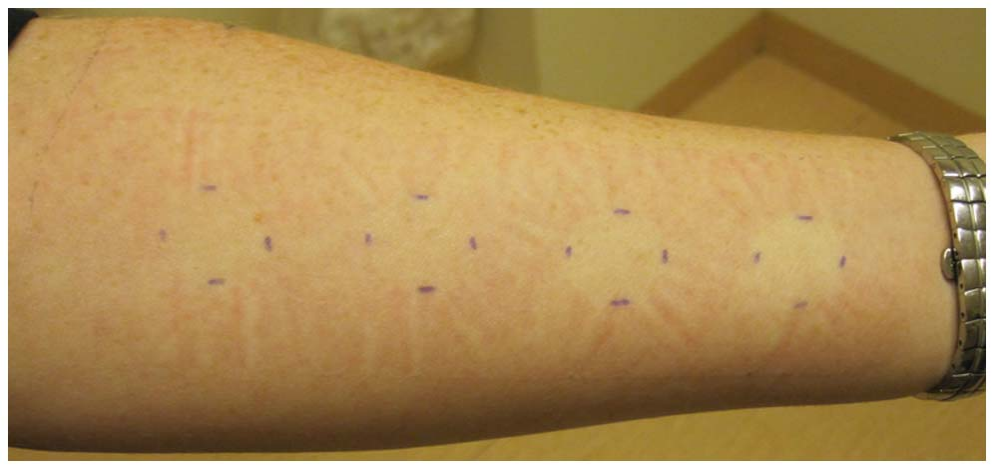
Blanching of the skin as a result of vasoconstriction, 7 hours after application of budesonide in increasing concentrations.

The corticosteroid skin blanching test was performed as described before [12]. Briefly, budesonide was dissolved in 95% ethanol to 8 logarithmically increasing concentrations: 0-0.33-1.0-3.3-10-33.3-100-333-1000 μg/ml. The different budesonide concentrations were randomly applied at 2 cm diameter test sites on the subject's forearm, 10 μl per test site. Sites were covered with plastic film. Assessment of blanching was performed after 7 hours by trained observers blinded for the concentration sequences using a 7-point scale, i.e. 0 (normal skin), 0.5, 1, 1.5, 2, 2.5, 3 (intense blanching) (Figure 1).

Pulmonary function and lung volumes were measured by spirometry and body plethysmography respectively, according to European Respiratory Society guidelines [14], [15].

### Statistical methods

Skin blanching response was expressed as the area under the dose-response curve (AUC) for each participant using the trapezoidal method [16]. Mann-Whitney U tests were used to compare skin blanching responses between groups, using the Benjamini-Hochberg correction for multiple testing [17]. Spearman's rank correlations were performed on skin blanching response and clinical characteristics. Multiple regression analyses were performed on skin blanching response with significant variables from univariate correlation analyses as predictor variables. We included lung function variables representing airway obstruction (FEV_1_, L), hyperinflation (RV/TLC, %), and small airway obstruction (MEF_50_, L/s). Since FEV_1_ and RV/TLC are highly correlated to each other, they were not entered in the model simultaneously. All models were adjusted for age, gender, and height. P-values <0.05 were considered significant. Data were analyzed using IBM SPSS Statistics version 20.

## Results

### Characteristics

We included 28 healthy never-smokers, 56 healthy smokers and 89 COPD patients; 27 GOLD stage I, 24 GOLD stage II, 22 GOLD stage III and 16 GOLD stage IV. Group characteristics are presented in Table 1. COPD patients had as higher age (p<0.01), were less frequently current smokers (p<0.01), and had a higher number of packyears (p<0.04) than healthy controls. As expected, lung function values were significantly lower (p<0.01) in COPD patients than in controls. In total 9 patients used oral corticosteroids (OCS). The number of patients using OCS was significantly higher (p = 0.03) in the severe-to-very severe (GOLD III+IV, n = 7) than in the mild-to-moderate patient group (GOLD I+II, n = 2).

**Table 1:** Characteristics of the study population.

	Healthy never-smokers	Healthy smokers	COPD GOLD I	COPD GOLD II	COPD GOLD III	COPD GOLD IV
**N**	28	56	27	24	22	16
**Male, n (%)**	20 (71)	41 (73)	22 (82)	18 (75)	16 (73)	7 (44)
**Age, years**	57 [51–66]	51 [46–58]	64 [58–68]	63 [59–70]	63 [56–65]	60 [53–66]
**Skin blanching, AUC**	1560 [1018–2180]	1154 [667–1771]	1380 [783–2481]	1309 [379–2189]	586 [17–1270]	243 [59–1385]
**Current smokers, n (%)**	0 (0)	55 (100)	18 (67)	16 (67)	11 (50)	3 (19)
**Cigarettes per day, n**	0	17 [11]–[20]	12 [6]–[23]	6 [4]–[15]	10 [4]–[20]	5 [5]–[6]
**Packyears**	0	26 [21–39]	40 [28–54]	31 [24–39]	42 [32–56]	31 [23–40]
**FEV_1_, L**	3.6 [3.1–4.4]	3.7 [3.3–4.2]	2.9 [2.7–3.4]	1.9 [1.7–2.1]	1.2 [1.1–1.4]	0.7 [0.6–0.8]
**FEV_1_/FVC, %**	79 [77–82]	78 [75–83]	64 [58–68]	50 [42–57]	38 [34–41]	31 [25–37]
**FEV_1_, % predicted**	111 [103–120]	107 [101–117]	95 [87–100]	66 [55–73]	41 [36–47]	25 [21]–[29]
**RV/TLC, %**	32 [27–35]	31 [28–34]	38 [32–41]	44 [38–49]	52 [47–57]	64 [55–66]
**MEF_50_, L/s**	4.0 [3.3–5.1]	4.0 [3.6–4.9]	2.0 [1.7–2.4]	0.8 [0.7–1.2]	0.4 [0.4–0.5]	0.3 [0.2–0.3]
**CS use:**	0 (0)	0 (0)	10 (37)	18 (75)	20 (91)	16 (100)
**ICS, n (%)**	0 (0)	0 (0)	9 (33)	17 (71)	19 (86)	10 (63)
**ICS, ug/ml per day** §			0 [0–500]	1000 [125–1750]	1000 [200–1000]	1000 [250–1188]
**OCS, n (%)**	0 (0)	0 (0)	1 (4)	1 (4)	1 (5)	6 (38)
**OCS, mg per day**			0 [0-0]	0 [0-0]	0 [0-0]	0 [0–5]

Data are expressed as median [Inter Quartile Range]. n =  number, AUC  =  area under the dose-response curve, FEV_1_ =  forced expiratory volume in one second, FVC =  forced vital capacity, RV =  residual volume, TLC =  total lung capacity, MEF_50_ =  maximal expiratory flow at 50% of vital capacity, CS =  Corticosteroids, ICS =  inhaled corticosteroids, OCS =  oral corticosteroids.

§ calculated as μg/day beclomethasone.

### Skin blanching response

**Figure 2:**
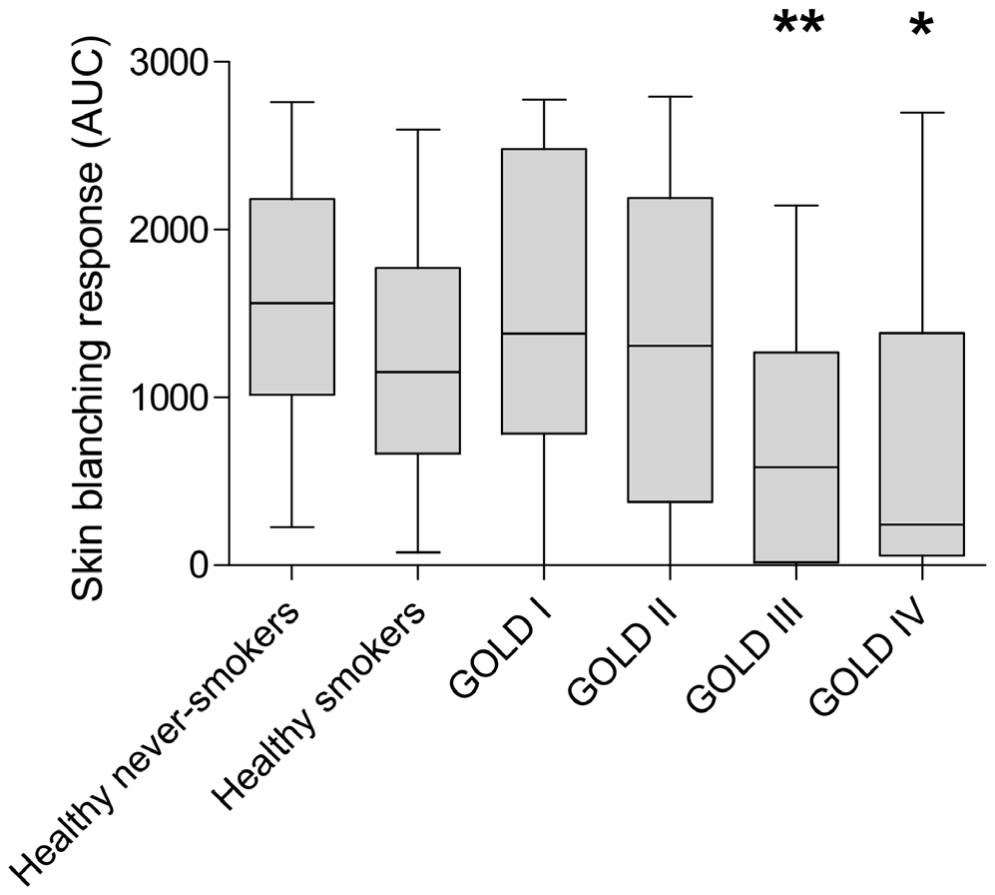
Skin blanching responses in healthy controls and COPD patients GOLD stages I–IV. AUC  =  area under the dose-response curve. Values are expressed as median [range]. ** significantly different from healthy never-smokers, healthy smokers, GOLD I and GOLD II patients (p-value <0.05) * significantly different from healthy never-smokers, healthy smokers and GOLD I patients (p-value ≤0.01)

**Figure 3:**
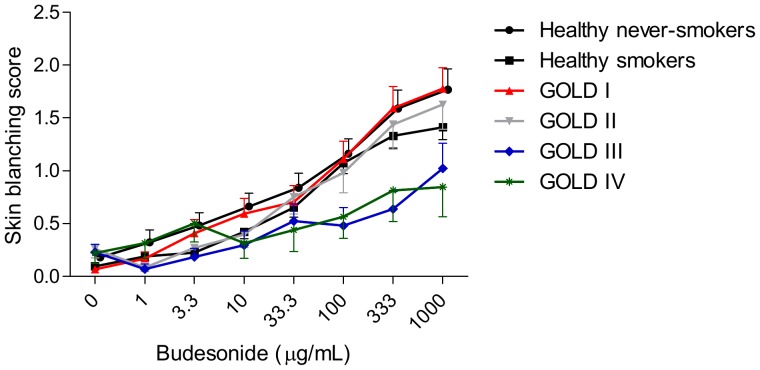
Dose-response curves of budesonide in healthy controls and COPD patients GOLD stages I–IV. Values are expressed as mean ± SEM.

COPD GOLD stage III patients had significantly lower skin blanching responses than healthy never-smokers and smokers (both p≤0.01), GOLD stage I (p<0.01) and GOLD stage II patients (p = 0.02) (Figures 2 and 3). GOLD stage IV patients had also significantly lower skin blanching responses than healthy never-smokers and smokers (both p<0.01) and GOLD stage I patients (p = 0.01), and had near significantly lower skin blanching responses than GOLD stage II patients (p = 0.06). The skin blanching response was comparable between healthy never-smokers and smokers and between patients with GOLD stages I and II, as were the responses between GOLD III and GOLD IV patients. Additionally, no significant differences in skin blanching responses were found between smoking and ex-smoking COPD patients (p>0.05).

### Predictors of skin blanching response

Table 2 presents results of monovariate correlations of the skin blanching response with gender, age, corticosteroid use, smoking parameters, and lung function. A lower skin blanching response was significantly correlated with female gender, corticosteroid use and reduced pulmonary function (lower FEV_1_, FEV_1_/FVC ratio, FEV_1_,%predicted, MEF_50_, and higher RV/TLC ratio)(p<0.05). The skin blanching response was not significantly correlated with age and smoking (current smoking, cigarettes per day and packyears).

**Table 2:** Univariate correlations of skin blanching response with clinical characteristics.

	N = 173	
	*Rho*	*p-value*
**Gender, male/female**	−0.177	**0.020**
**Age, years**	−0.035	0.644
**Corticosteroid use, no/yes**	−0.196	**0.010**
**Current smoking, no/yes**	−0.092	0.226
**Cigarettes per day, n**	0.073	0.469
**Packyears**	−0.107	0.162
**FEV_1_, L**	0.329	**0.000**
**FEV_1_/FVC, %**	0.215	**0.004**
**FEV_1_, %predicted**	0.247	**0.001**
**MEF_50_, L/s**	0.257	**0.001**
**RV/TLC, %**	−0.299	**0.000**

Spearman's rank correlations with skin blanching response (AUC) as dependent variable.

FEV_1_ =  forced expiratory volume in one second, FVC =  forced vital capacity, RV =  residual volume, TLC =  total lung capacity, MEF_50_ =  maximal expiratory flow at 50% of vital capacity, TLCO =  carbon monoxide transfer factor, VA =  accessible lung volume.

Values in bold represent significant correlations (p-value <0.05).

Table 3 presents the results of the two multivariate regression models. A lower skin blanching response was significantly associated with a lower FEV_1_ (p<0.01), and with a higher RV/TLC ratio (p<0.01). Effects of CS use in both models remained the same and R-square values of both models were comparable, 0.144 and 0.118 respectively. No significant associations were present between skin blanching response and MEF_50_. Importantly, corticosteroid use was not a predictor of skin blanching response in both multiple regression models.

**Table 3:** Linear regression analyses on skin blanching response (AUC).

	Model 1			Model 2		
N = 173	R^2^ = 0.144			R^2^ = 0.118		
	B	S.E.	*p*-value	B	S.E.	*p*-value
**FEV_1_, L**	490.9	140.8	**0.001**	---	---	---
**RV/TLC, %**	---	---	---	−25.4	2.8	**0.004**
**MEF_50_, L/s**	−130.1	78.5	0.099	−17.4	58.6	0.767
**Corticosteroid use, no/yes**	218.7	209.5	0.298	115.4	210.0	0.583

Since FEV_1_ and RV/TLC are highly correlated to each other, they were not entered in the model simultaneously. Dependent variable is skin blanching response (AUC). Both models were corrected for age, gender, and height. B =  regression coefficient, SE  =  standard error, FEV_1_ =  forced expiratory volume in one second, FVC  =  forced vital capacity, RV  =  residual volume, TLC  =  total lung capacity, MEF_50_  =  maximal expiratory flow at 50% of vital capacity, TLCO  =  carbon monoxide transfer factor, VA  =  accessible lung volume. Values in bold represent significant p-values (< 0.05).

### Effects of corticosteroid treatment on the skin blanching response

**Figure 4:**
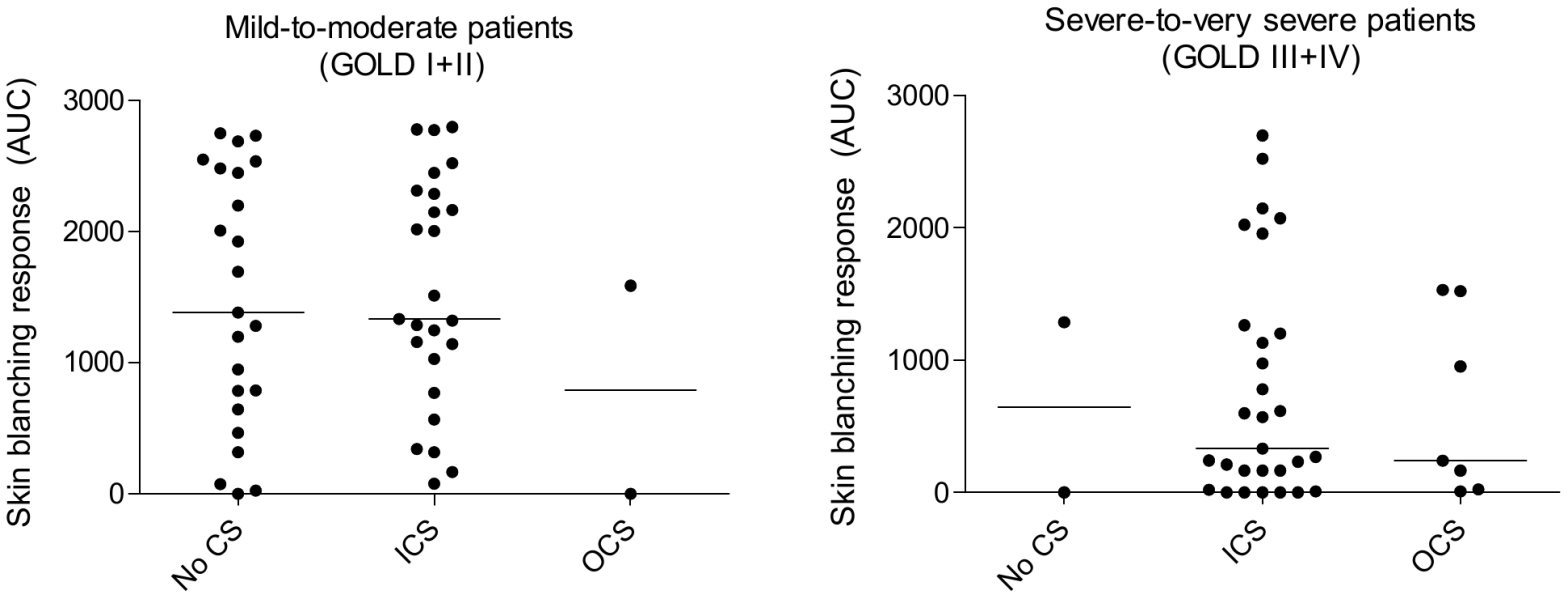
Corticosteroid treatment and skin blanching response in COPD patients. AUC  =  area under the dose-response curve, CS  =  corticosteroids, ICS  =  inhaled corticosteroids, OCS  =  oral corticosteroids.

In the GOLD I+II patient group, 26 patients used ICS treatment, 2 patients OCS, and 23 patients used no corticosteroids (Table 1). In the GOLD III+IV patient group, numbers were respectively 29, 7, and 2 (Table 1). COPD patients with and without ICS had similar median [IQR] skin blanching responses as determined by AUC values, both in the GOLD I and II groups (1327 [718–2293] and 1380 [640–2480] respectively), and the GOLD III and IV groups (333 [95–1231] and 645 [2–645] respectively) (Figure 4). The same was found for OCS, median [IQR] AUC values being 795 [2.37–795] and 241 [23–1520] in the GOLD I+II and GOLD III+IV groups respectively.

## Discussion

The current study shows that the skin blanching response to topical budesonide is lower in patients with more severe COPD. Patients with COPD GOLD stage III and IV had significantly lower skin blanching response than those with stage I and II and the latter groups had a response similar to that in healthy never- and current-smokers. These observations were not driven by corticosteroid use. In line, a lower skin blanching response was associated with a lower FEV_1_ and a higher RV/TLC ratio in multivariate regression analyses, and not affected by smoking. These data indicate that reduced corticosteroid sensitivity is present in a sub-population of COPD patients.

The association we found between FEV_1_ and skin blanching response in COPD is consistent with observations from our earlier study in asthma, in which patients with an FEV_1_<80% predicted had a lower skin blanching response than those without airway obstruction [12]. Our findings are also in line with Brown et al, showing that corticosteroid resistant asthma patients had lower skin blanching scores than steroid sensitive patients [10]. This may be due to their steroid resistance, but compatible with our observation steroid resistant patients had also lower FEV_1_%predicted values than steroid sensitive patients. In our COPD population we additionally focused on the relation between skin blanching response and lung function parameters representing hyperinflation and small airway obstruction. Monovariate analyses showed that besides a lower FEV_1_, lower skin blanching response correlated significantly with a lower MEF_50_ and more hyperinflation (RV/TLC). Of these parameters, FEV_1_ and RV/TLC ratio appeared to be the most important predictors of the skin blanching response, since both remained significant in multiple linear regression analyses. These are novel observations which have not been described before. Taken together, our data suggest an interesting link between a lower skin blanching response and worse lung function.

The question arises whether the observed impaired corticosteroid sensitivity originated during the progression of COPD, or whether it was already present earlier in life, possibly contributing to the development of COPD. In this context, our findings that mild and moderately severe COPD patients had skin blanching responses which were comparable to those of healthy controls are of interest. This finding makes it less likely that a reduced corticosteroid sensitivity originates as a result of COPD development. Importantly, all COPD patient groups (GOLD I, II, III and IV) were of comparable age and had smoked the same high number of packyears. However, despite similar age and packyears, main determinants of lung function level, patients with GOLD stage III and IV had excessively lower lung function values. This suggests that they were more susceptible to the detrimental effects of smoking and possibly suffer from a different type of COPD e.g. by early accelerated lung function decline resulting in early-onset of COPD [18], [19]. We therefore hypothesize that the skin blanching response may have discriminative property to point at a subphenotype of COPD by recognizing patients with an accelerated loss of lung function at an earlier stage in life.

Phenotyping of COPD patients aims to identify subgroups of patients, based on clinically important characteristics, which could be useful to better understand the origin of COPD and to optimize treatment. Traditionally, distinctions have been made between patients with emphysema and chronic bronchitis, two clinical phenotypes which in most cases are both present to a varying extent [20]. We found no indications that corticosteroid insensitivity is related with those phenotypes, since skin blanching response was not associated with diffusion capacity and symptoms (phlegm production and shortness of breath, data not shown). Several phenotypes of COPD have been proposed, based on outcomes which may vary greatly between patients, e.g. clinical and physiological manifestations, radiologic characterization, exacerbation frequency, systemic inflammation, and comorbidities [21]. Since COPD patients show individually different benefits to corticosteroids, this suggests that a corticosteroid-responsive phenotype could also be present. If we assume that the response in the skin can be translated to the airways, the skin blanching response might differentiate between those responders and non-responders to corticosteroid treatment. This clearly needs further prospective study.

There are indications that corticosteroid insensitivity is genetically determined. The skin blanching response has been shown to be regulated by vascular smooth muscle glucocorticoid receptors (GR) [22]–[24], leading to local vasoconstriction [25]. Studies investigating the association between GR single nucleotide polymorphisms (SNPs) and corticosteroid sensitivity have shown that individuals with a *BclI* SNP express a lower skin blanching response [24], [26]. This SNP may result in different GR isoform expression, receptor affinity or GR expression. The change in GR function that affects corticosteroid response is of putative importance, since endogenous corticosteroids are essential to normal lung development during fetal growth [27]. Thus, small changes in corticosteroid sensitivity may have important consequences for lung development and lung growth in utero and early childhood. It has been shown that lower lung function at birth or in early childhood increases the risk of COPD development [18]. Furthermore, corticosteroid insensitivity may associate with a higher susceptibility to harmful external stimuli, like cigarette smoking and air pollution. The subsequently enhanced inflammatory condition may contribute to further remodeling and fibrosis of the airways and/or emphysematous changes in lung tissue together resulting in airway obstruction [28]. In this way our findings of reduced corticosteroid sensitivity in a subgroup of COPD patients may reflect an underlying genetic contribution to an early onset COPD phenotype [29].

Patients with severe COPD use more often corticosteroid treatment compared to milder stages of COPD, in line with the GOLD guidelines that advocate ICS in this patient category [1]. Also in the current study corticosteroid use was higher prevalent in GOLD stage III and IV patients. It is of importance to realize that corticosteroid treatment may interfere with the GR receptor, thereby affecting the skin blanching response. We observed no differences in skin blanching response between patients using ICS and/or OCS, and patients using no corticosteroids at all. Monovariate correlation analyses showed that corticosteroid use was correlated with a lower skin blanching response. However, corticosteroid use was no predictor of skin blanching response in multiple regression models, demonstrating the more important role of lung function as predictor of skin blanching. In addition, we have investigated the effects of beta-2 agonists and anticholinergics on the skin blanching response, since these drugs can affect vasoconstriction responses in humans [30]. We did not find a significant association between both drugs and the skin blanching response (data not shown).

In conclusion, the current study shows that severe and very severe COPD patients (GOLD III and IV) have lower corticosteroid sensitivity in the skin compared with mild and moderate COPD patients (GOLD I and II) and healthy never-smokers and smokers. It remains speculative which mechanisms drive this observation. We put forward the hypothesis that corticosteroid insensitivity is a genetically determined phenotype which may contribute to the development of COPD. To further investigate, validate and extend our findings, a next step would be to replicate this research in future studies, expanding the group size and associating skin blanching response with differential genetic profiles in a population of COPD patients and healthy controls. This would gain more insight in different COPD phenotypes and may be helpful in understanding the corticosteroid resistant phenotype that can be present in COPD.
